# Bioconductor: open software development for computational biology and bioinformatics

**DOI:** 10.1186/gb-2004-5-10-r80

**Published:** 2004-09-15

**Authors:** Robert C Gentleman, Vincent J Carey, Douglas M Bates, Ben Bolstad, Marcel Dettling, Sandrine Dudoit, Byron Ellis, Laurent Gautier, Yongchao Ge, Jeff Gentry, Kurt Hornik, Torsten Hothorn, Wolfgang Huber, Stefano Iacus, Rafael Irizarry, Friedrich Leisch, Cheng Li, Martin Maechler, Anthony J Rossini, Gunther Sawitzki, Colin Smith, Gordon Smyth, Luke Tierney, Jean YH Yang, Jianhua Zhang

**Affiliations:** 1 Department of Biostatistical Science, Dana-Farber Cancer Institute, 44 Binney St, Boston, MA 02115, USA; 2 Channing Laboratory, Brigham and Women's Hospital, 75 Francis Street, Boston, MA 02115, USA; 3 Department of Statistics, University of Wisconsin-Madison, 1210 W Dayton St, Madison, WI 53706, USA; 4 Division of Biostatistics, University of California, Berkeley, 140 Warren Hall, Berkeley, CA 94720-7360, USA; 5 Seminar for Statistics LEO C16, ETH Zentrum, Zürich CH-8092, Switzerl; 6 Department of Statistics, Harvard University, 1 Oxford St, Cambridge, MA 02138, USA; 7 Center for Biological Sequence Analysis, Technical University of Denmark, Building 208, Lyngby 2800, Denmark; 8 Department of Biomathematical Sciences, Mount Sinai School of Medicine, 1 Gustave Levy Place, Box 1023, New York, NY 10029, USA; 9 Institut für Statistik und Wahrscheinlichkeitstheorie, TU Wien, Wiedner Hauptstrasse 8-10/1071, Wien 1040, Austria; 10 Institut für Medizininformatik, Biometrie und Epidemiologie, Friedrich-Alexander-Universität Erlangen-Nürnberg, Waldstraße6, D-91054 Erlangen, Germany; 11 Division of Molecular Genome Analysis, DKFZ (German Cancer Research Center), 69120 Heidelberg, Germany; 12 Department of Economics, University of Milan, 23 Via Mercalli, I-20123 Milan, Italy; 13 Department of Biostatistics, Johns Hopkins University, 615 N Wolfe St E3035, Baltimore, MD 21205, USA; 14 Department of Medical Education and Biomedical Informatics, University of Washington, Box 357240, 1959 NE Pacific, Seattle, WA 98195, USA; 15 Statistisches Labor, Institut für Angewandte Mathematik, Im Neuenheimer Feld 294, D 69120, Heidelberg, Germany; 16 Department of Molecular Biology, The Scripps Research Institute, 10550 North Torrey Pines Road, TPC-28, La Jolla, CA 92037, USA; 17 Division of Genetics and Bioinformatics, The Walter and Eliza Hall Institute of Medical Research, 1G Royal Parade, Parkville, Victoria 3050, Australia; 18 Department of Statistics and Actuarial Science, University of Iowa, 241 Schaeffer Hall, Iowa City, IA 52242, USA; 19 Center for Bioinformatics and Molecular Biostatistics, Univerisity of California, San Francisco, 500 Parnassus Ave, San Francisco 94143-0560, USA

## Abstract

The Bioconductor project is an initiative for the collaborative creation of extensible software for computational biology and bioinformatics. The goals of the project include: fostering collaborative development and widespread use of innovative software, reducing barriers to entry into interdisciplinary scientific research, and promoting the achievement of remote reproducibility of research results. We describe details of our aims and methods, identify current challenges, compare Bioconductor to other open bioinformatics projects, and provide working examples.

## Abstract 2

A detailed description of the aims and methods of the Bioconductor project, an initiative for the collaborative creation of extensible software for computational biology and bioinformatics.

## Background

The Bioconductor project [1] is an initiative for the collaborative creation of extensible software for computational biology and bioinformatics (CBB). Biology, molecular biology in particular, is undergoing two related transformations. First, there is a growing awareness of the computational nature of many biological processes and that computational and statistical models can be used to great benefit. Second, developments in high-throughput data acquisition produce requirements for computational and statistical sophistication at each stage of the biological research pipeline. The main goal of the Bioconductor project is creation of a durable and flexible software development and deployment environment that meets these new conceptual, computational and inferential challenges. We strive to reduce barriers to entry to research in CBB. A key aim is simplification of the processes by which statistical researchers can explore and interact fruitfully with data resources and algorithms of CBB, and by which working biologists obtain access to and use of state-of-the-art statistical methods for accurate inference in CBB.

Among the many challenges that arise for both statisticians and biologists are tasks of data acquisition, data management, data transformation, data modeling, combining different data sources, making use of evolving machine learning methods, and developing new modeling strategies suitable to CBB. We have emphasized transparency, reproducibility, and efficiency of development in our response to these challenges. Fundamental to all these tasks is the need for software; ideas alone cannot solve the substantial problems that arise.

The primary motivations for an open-source computing environment for statistical genomics are transparency, pursuit of reproducibility and efficiency of development.

### Transparency

High-throughput methodologies in CBB are extremely complex, and many steps are involved in the conversion of information from low-level information structures (for example, microarray scan images) to statistical databases of expression measures coupled with design and covariate data. It is not possible to say *a priori *how sensitive the ultimate analyses are to variations or errors in the many steps in the pipeline. Credible work in this domain requires exposure of the entire process.

### Pursuit of reproducibility

Experimental protocols in molecular biology are fully published lists of ingredients and algorithms for creating specific substances or processes. Accuracy of an experimental claim can be checked by complete obedience to the protocol. This standard should be adopted for algorithmic work in CBB. Portable source code should accompany each published analysis, coupled with the data on which the analysis is based.

### Efficiency of development

By development, we refer not only to the development of the specific computing resource but to the development of computing methods in CBB as a whole. Software and data resources in an open-source environment can be read by interested investigators, and can be modified and extended to achieve new functionalities. Novices can use the open sources as learning materials. This is particularly effective when good documentation protocols are established. The open-source approach thus aids in recruitment and training of future generations of scientists and software developers.

The rest of this article is devoted to describing the computing science methodology underlying Bioconductor. The main sections detail design methods and specific coding and deployment approaches, describe specific unmet challenges and review limitations and future aims. We then consider a number of other open-source projects that provide software solutions for CBB and end with an example of how one might use Bioconductor software to analyze microarray data.

## Results and discussion

### Methodology

The software development strategy we have adopted has several precedents. In the mid-1980s Richard Stallman started the Free Software Foundation and the GNU project [2] as an attempt to provide a free and open implementation of the Unix operating system. One of the major motivations for the project was the idea that for researchers in computational sciences "their creations/discoveries (software) should be available for everyone to test, justify, replicate and work on to boost further scientific innovation" [3]. Together with the Linux kernel, the GNU/Linux combination sparked the huge open-source movement we know today. Open-source software is no longer viewed with prejudice, it has been adopted by major information technology companies and has changed the way we think about computational sciences. A large body of literature exists on how to manage open-source software projects: see Hill [4] for a good introduction and a comprehensive bibliography.

One of the key success factors of the Linux kernel is its modular design, which allows for independent and parallel development of code [5] in a virtual decentralized network [3]. Developers are not managed within the hierarchy of a company, but are directly responsible for parts of the project and interact directly (where necessary) to build a complex system [6]. Our organization and development model has attempted to follow these principles, as well as those that have evolved from the R project [7,8].

In this section, we review seven topics important to establishment of a scientific open source software project and discuss them from a CBB point of view: language selection, infrastructure resources, design strategies and commitments, distributed development and recruitment of developers, reuse of exogenous resources, publication and licensure of code, and documentation.

#### Language selection

CBB poses a wide range of challenges, and any software development project will need to consider which specific aspects it will address. For the Bioconductor project we wanted to focus initially on bioinformatics problems. In particular we were interested in data management and analysis problems associated with DNA microarrays. This orientation necessitated a programming environment that had good numerical capabilities, flexible visualization capabilities, access to databases and a wide range of statistical and mathematical algorithms. Our collective experience with R suggested that its range of well-implemented statistical and visualization tools would decrease development and distribution time for robust software for CBB. We also note that R is gaining widespread usage within the CBB community independently of the Bioconductor Project. Many other bioinformatics projects and researchers have found R to be a good language and toolset with which to work. Examples include the Spot system [9], MAANOVA [10] and dChip [11]. We now briefly enumerate features of the R software environment that are important motivations behind its selection.

##### Prototyping capabilities

R is a high-level interpreted language in which one can easily and quickly prototype new computational methods. These methods may not run quickly in the interpreted implementation, and those that are successful and that get widely used will often need to be re-implemented to run faster. This is often a good compromise; we can explore lots of concepts easily and put more effort into those that are successful.

##### Packaging protocol

The R environment includes a well established system for packaging together related software components and documentation. There is a great deal of support in the language for creating, testing, and distributing software in the form of 'packages'. Using a package system lets us develop different software modules and distribute them with clear notions of protocol compliance, test-based validation, version identification, and package interdependencies. The packaging system has been adopted by hundreds of developers around the world and lies at the heart of the Comprehensive R Archive Network, where several hundred independent but interoperable packages addressing a wide range of statistical analysis and visualization objectives may be downloaded as open source.

##### Object-oriented programming support

The complexity of problems in CBB is often translated into a need for many different software tools to attack a single problem. Thus, many software packages are used for a single analysis. To secure reliable package interoperability, we have adopted a formal object-oriented programming discipline, as encoded in the 'S4' system of formal classes and methods [12]. The Bioconductor project was an early adopter of the S4 discipline and was the motivation for a number of improvements (established by John Chambers) in object-oriented programming for R.

##### WWW connectivity

Access to data from on-line sources is an essential part of most CBB projects. R has a well developed and tested set of functions and packages that provide access to different databases and to web resources (via http, for example). There is also a package for dealing with XML [13], available from the Omegahat project, and an early version of a package for a SOAP client [14], SSOAP, also available from the Omegahat project. These are much in line with proposals made by Stein [15] and have aided our work towards creating an environment in which the user perceives tight integration of diverse data, annotation and analysis resources.

##### Statistical simulation and modeling support

Among the statistical and numerical algorithms provided by R are its random number generators and machine learning algorithms. These have been well tested and are known to be reliable. The Bioconductor Project has been able to adapt these to the requirements in CBB with minimal effort. It is also worth noting that a number of innovations and extensions based on work of researchers involved in the Bioconductor project have been flowing back to the authors of these packages.

##### Visualization support

Among the strengths of R are its data and model visualization capabilities. Like many other areas of R these capabilities are still evolving. We have been able to quickly develop plots to render genes at their chromosomal locations, a heatmap function, along with many other graphical tools. There are clear needs to make many of these plots interactive so that users can query them and navigate through them and our future plans involve such developments.

##### Support for concurrent computation

R has also been the basis for pathbreaking research in parallel statistical computing. Packages such as *snow *and *rpvm *simplify the development of portable interpreted code for computing on a Beowulf or similar computational cluster of workstations. These tools provide simple interfaces that allow for high-level experimentation in parallel computation by computing on functions and environments in concurrent R sessions on possibly heterogeneous machines. The *snow *package provides a higher level of abstraction that is independent of the communication technology such as the message-passing interface (MPI) [16] or the parallel virtual machine (PVM) [17]. Parallel random number generation [18], essential when distributing parts of stochastic simulations across a cluster, is managed by *rsprng*. Practical benefits and problems involved with programming parallel processes in R are described more fully in Rossini *et al. *[19] and Li and Rossini [20].

##### Community

Perhaps the most important aspect of using R is its active user and developer communities. This is not a static language. R is undergoing major changes that focus on the changing technological landscape of scientific computing. Exposing biologists to these innovations and simultaneously exposing those involved in statistical computing to the needs of the CBB community has been very fruitful and we hope beneficial to both communities.

#### Infrastructure base

We began with the perspective that significant investment in software infrastructure would be necessary at the early stages. The first two years of the Bioconductor project have included significant effort in developing infrastructure in the form of reusable data structures and software/documentation modules (R packages). The focus on reusable software components is in sharp contrast to the one-off approach that is often adopted. In a one-off solution to a bioinformatics problem, code is written to obtain the answer to a given question. The code is not designed to work for variations on that question or to be adaptable for application to distinct questions, and may indeed only work on the specific dataset to which it was originally applied. A researcher who wishes to perform a kindred analysis must typically construct the tools from scratch. In this situation, the scientific standard of reproducibility of research is not met except via laborious reinvention. It is our hope that reuse, refinement and extension will become the primary software-related activities in bioinformatics. When reusable components are distributed on a sound platform, it becomes feasible to demand that a published novel analysis be accompanied by portable and open software tools that perform all the relevant calculations. This will facilitate direct reproducibility, and will increase the efficiency of research by making transparent the means to vary or extend the new computational method.

Two examples of the software infrastructure concepts described here are the exprSet class of the *Biobase *package, and the various Bioconductor metadata packages, for example *hgu95av2*. An exprSet is a data structure that binds together array-based expression measurements with covariate and administrative data for a collection of microarrays. Based on R data.frame and list structures, exprSets offer much convenience to programmers and analysts for gene filtering, constructing annotation-based subsets, and for other manipulations of microarray results. The exprSet design facilitates a three-tier architecture for providing analysis tools for new microarray platforms: low-level data are bridged to high-level analysis manipulations via the exprSet structure. The designer of low-level processing software can focus on the creation of an exprSet instance, and need not cater for any particular analysis data structure representation. The designer of analysis procedures can ignore low-level structures and processes, and operate directly on the exprSet representation. This design is responsible for the ease of interoperation of three key Bioconductor packages: *affy*, *marray*, and *limma*.

The *hgu95av2 *package is one of a large collection of related packages that relate manufactured chip components to biological metadata concerning sequence, gene functionality, gene membership in pathways, and physical and administrative information about genes. The package includes a number of conventionally named hashed environments providing high-performance retrieval of metadata based on probe nomenclature, or retrieval of groups of probe names based on metadata specifications. Both types of information (metadata and probe name sets) can be used very fruitfully with exprSets: for example, a vector of probe names immediately serves to extract the expression values for the named probes, because the exprSet structure inherits the named extraction capacity of R data.frames.

#### Design strategies and commitments

Well-designed scientific software should reduce data complexity, ease access to modeling tools and support integrated access to diverse data resources at a variety of levels. Software infrastructure can form a basis for both good scientific practice (others should be able to easily replicate experimental results) and for innovation.

The adoption of designing by contract, object-oriented programming, modularization, multiscale executable documentation, and automated resource distribution are some of the basic software engineering strategies employed by the Bioconductor Project.

##### Designing by contract

While we do not employ formal contracting methodologies (for example, Eiffel [21]) in our coding disciplines, the contracting metaphor is still useful in characterizing the approach to the creation of interoperable components in Bioconductor. As an example, consider the problem of facilitating analysis of expression data stored in a relational database, with the constraints that one wants to be able to work with the data as one would with any exprSet and one does not want to copy unneeded records into R at any time. Technically, data access could occur in various ways, using database connections, DCOM [22], communications or CORBA [23], to name but a few. In a designing by contract discipline, the provider of exprSet functionality must deliver a specified set of functionalities. Whatever object the provider's code returns, it must satisfy the exprSets contract. Among other things, this means that the object must respond to the application of functions exprs and pData with objects that satisfy the R matrix and data.frame contracts respectively. It follows that exprs(*x*) [*i,j*], for example, will return the number encoding the expression level for the *i*th gene for the *j*th sample in the object *x*, no matter what the underlying representation of *x*. Here *i *and *j *need not denote numerical indices but can hold any vectors suitable for interrogating matrices via the square-bracket operator. Satisfaction of the contract obligations simplifies specification of analysis procedures, which can be written without any concern for the underlying representations for exprSet information.

A basic theme in R development is simplifying the means by which developers can state, follow, and verify satisfaction of design contracts of this sort. Environment features that support convenient inheritance of behaviors between related classes with minimal recoding are at a premium in this discipline.

##### Object-oriented programming

There are various approaches to the object-oriented programming methodology. We have encouraged, but do not require, use of the so-called S4 system of formal classes and methods in Bioconductor software. The S4 object paradigm (defined primarily by Chambers [12] with modifications embodied in R) is similar to that of Common Lisp [24] and Dylan [25]. In this system, classes are defined to have specified structures (in terms of a set of typed 'slots') and inheritance relationships, and methods are defined both generically (to specify the basic contract and behavior) and specifically (to cater for objects of particular classes). Constraints can be given for objects intended to instantiate a given class, and objects can be checked for validity of contract satisfaction. The S4 system is a basic tool in carrying out the designing by contract discipline, and has proven quite effective.

##### Modularization

The notion that software should be designed as a system of interacting modules is fairly well established. Modularization can occur at various levels of system structure. We strive for modularization at the data structure, R function and R package levels. This means that data structures are designed to possess minimally sufficient content to have a meaningful role in efficient programming. The exprSet structure, for example, contains information on expression levels (exprs slot), variability (se.exprs), covariate data (phenoData slot), and several types of metadata (slots description, annotation and notes). The tight binding of covariate data with expression data spares developers the need to track these two types of information separately. The exprSet structure explicitly excludes information on gene-related annotation (such as gene symbol or chromosome location) because these are potentially volatile and are not needed in many activities involving exprSets. Modularization at the R function level entails that functions are written to do one meaningful task and no more, and that documents (help pages) are available at the function level with worked examples. This simplifies debugging and testing. Modularization at the package level entails that all packages include sufficient functionality and documentation to be used and understood in isolation from most other packages. Exceptions are formally encoded in files distributed with the package.

##### Multiscale and executable documentation

Accurate and thorough documentation is fundamental to effective software development and use, and must be created and maintained in a uniform fashion to have the greatest impact. We inherit from R a powerful system for small-scale documentation and unit testing in the form of the executable example sections in function-oriented manual pages. We have also introduced a new concept of large-scale documentation with the *vignette *concept. Vignettes go beyond typical man page documentation, which generally focuses on documenting the behavior of a function or small group of functions. The purpose of a vignette is to describe in detail the processing steps required to perform a specific task, which generally involves multiple functions and may involve multiple packages. Users of a package have interactive access to all vignettes associated with that package.

The *Sweave *system [26] was adopted for creating and processing vignettes. Once these have been written users can interact with them on different levels. The transformed documents are provided in Adobe's portable document format (PDF) and access to the code chunks from within R is available through various functions in the *tools *package. However, new users will need a simpler interface. Our first offering in this area is the vignette explorer vExplorer which provides a widget that can be used to navigate the various code chunks. Each chunk is associated with a button and the code is displayed in a window, within the widget. When the user clicks on the button the code is evaluated and the output presented in a second window. Other buttons provide other functionality, such as access to the PDF version of the document. We plan to extend this tool greatly in the coming years and to integrate it closely with research into reproducible research (see [27] for an illustration).

##### Automated software distribution

The modularity commitment imposes a cost on users who are accustomed to integrated 'end-to-end' environments. Users of Bioconductor need to be familiar with the existence and functionality of a large number of packages. To diminish this cost, we have extended the packaging infrastructure of R/CRAN to better support the deployment and management of packages at the user level. Automatic updating of packages when new versions are available and tools that obtain all package dependencies automatically are among the features provided as part of the reposTools package in Bioconductor. Note that new methods in R package design and distribution include the provision of MD5 checksums with all packages, to help with verification that package contents have not been altered in transit.

In conclusion, these engineering commitments and developments have led to a reasonably harmonious set of tools for CBB. It is worth considering how the S language notion that 'everything is an object' impacts our approach. We have made use of this notion in our commitment to contracting and object-oriented programming, and in the automated distribution of resources, in which package catalogs and biological metadata are all straightforward R objects. Packages and documents are not yet treatable as R objects, and this leads to complications. We are actively studying methods for simplifying authoring and use of documentation in a multipackage environment with namespaces that allow symbol reuse, and for strengthening the connection between session image and package inventory in use, so that saved R images can be restored exactly to their functional state at session close.

#### Distributed development and recruitment of developers

Distributed development is the process by which individuals who are significantly geographically separated produce and extend a software project. This approach has been used by the R project for approximately 10 years. This was necessitated in this case by the fact no institution currently has sufficient numbers of researchers in this area to support a project of this magnitude. Distributed development facilitates the inclusion of a variety of viewpoints and experiences. Contributions from individuals outside the project led to the expansion of the core developer group. Membership in the core depends upon the willingness of the developer to adopt shared objectives and methods and to submerge personal objectives in preference to creation of software for the greater scientific community.

Distributed development requires the use of tools and strategies that allow different programmers to work approximately simultaneously on the same components of the project. Among the more important requirements is for a shared code base (or archive) that all members of the project can access and modify together with some form of version management system. We adopted the Concurrent Versions System [28,29] and created a central archive, within this system, that all members of the team have access to.

Additional discipline is needed to ensure that changes by one programmer should not result in a failure of other code in the system. Within the R language, software components are naturally broken into packages, with a formal protocol for package structure and content specified in the R Extensions manual [30]. Each package should represent a single coherent theme. By using well defined applications programming interfaces (APIs) developers of a package are free to modify their internal structures as long as they continue to provide the documented outputs.

We rely on the testing mechanisms supported by the R package testing system [30] to ensure coherent, non-regressive development. Each developer is responsible for documenting all functions and for providing examples and possibly other scripts or sets of commands that test the code. Each developer is responsible for ensuring that all tests run successfully before committing changes back to the central archive. Thus, the person who knows the code best writes the test programs, but all are responsible for running them and ensuring that changes they have made do not affect the code of others. In some cases changes by one author will necessitate change in the code and tests of others. Under the system we are using these situations are detected and dealt with when they occur in development, reducing the frequency with which error reports come from the field.

Members of the development team communicate via a private mailing list. In many cases they also use private email, telephone and meetings at conferences in order to engage in joint projects and to keep informed about the ideas of other members.

#### Reuse of exogenous resources

We now present three arguments in favor of using and adapting software from other projects rather than re-implementing or reinventing functionality. The first argument that we consider is that writing good software is a challenging problem and any re-implementation of existing algorithms should be avoided if possible. Standard tools and paradigms that have been proven and are well understood should be preferred over new untested approaches. All software contains bugs but well used and maintained software tends to contain fewer.

The second argument is that CBB is an enormous field and that progress will require the coordinated efforts of many projects and software developers. Thus, we will require structured paradigms for accessing data and algorithms written in other languages and systems. The more structured and integrated this functionality, the easier it will be to use and hence the more it will be used. As specific examples we consider our recent development of tools for working with graph or network structures. There are three main packages in Bioconductor of interacting with graphs. They are *graph*, *RBGL *and *Rgraphviz*. The first of these provides the class descriptions and basic infrastructure for dealing with graphs in R, the second provides access to algorithms on graphs, and the third to a rich collection of graph layout algorithms. The *graph *package was written from scratch for this project, but the other two are interfaces to rich libraries of software routines that have been created by other software projects, BOOST [31,32] and *Graphviz *[23] respectively, both of which are very substantial projects with large code bases. We have no interest in replicating that work and will, wherever possible, simply access the functions and libraries produced by other projects.

There are many benefits from this approach for us and for the other projects. For bioinformatics and computational biology we gain rapid access to a variety of graph algorithms including graph layout and traversal. The developers in those communities gain a new user base and a new set of problems that they can consider. Gaining a new user base is often very useful, as new users with previously unanticipated needs tend to expose weaknesses in design and implementation that more sophisticated or experienced users are often able to avoid.

In a similar vein, we plan to develop and encourage collaboration with other projects, including those organized through the Open Bioinformatics Foundation and the International Interoperability Consortium. We have not specifically concentrated on collaboration to this point in part because we have chosen areas for development that do not overlap significantly with the tools provided by those projects. In this case our philosophy remains one of developing interfaces to the software provided by those projects and not re-implementing their work. In some cases, other projects have recognized the potential gains for collaboration and have started developing interfaces for us to their systems, with the intent of making future contributions [33].

Another argument in favor of standardization and reuse of existing tools is best made with reference to a specific example. Consider the topic of markup and markup languages. For any specific problem one could quickly devise a markup that is sufficient for that problem. So why then should we adopt a standard such as XML? Among the reasons for this choice is the availability of programmers conversant with the paradigm, and hence lower training costs. A second reason is that the XML community is growing and developing and we will get substantial technological improvements without having to initiate them. This is not unusual. Other areas of computational research are as vibrant as CBB and by coordinating and sharing ideas and innovations we simplify our own tasks while providing stimulus to these other areas.

#### Publication and licensing of code

Modern standards of scientific publication involve peer review and subsequent publication in a journal. Software publication is a slightly different process with limited involvement to date of formal peer review or official journal publication. We release software under an open-source license as our main method of publication. We do this in the hope that it will encourage reproducibility, extension and general adherence to the scientific method. This decision also ensures that the code is open to public scrutiny and comment. There are many other reasons for deciding to release software under an open-source license, some of which are listed in Table 1.

Another consideration that arose when determining the form of publication was the need to allow an evolutionary aspect to our own software. There are many reasons for adopting a strategy that would permit us to extend and improve our software offerings over time. The field of CBB is relatively volatile and as new technologies are developed new software and inferential methods are needed. Further, software technology itself is evolving. Thus, we wanted to have a publication strategy that could accommodate changes in software at a variety of levels. We hope that that strategy will also encourage our users to think of software technology as a dynamic field rather than a static one and to therefore be on the lookout for innovations in this arena as well as in more traditional biological ones.

Our decision to release software in the form of R packages is an important part of this consideration. Packages are easy to distribute, they have version numbers and define an API. A coordinated release of all Bioconductor packages occurs twice every year. At any given time there is a release version of every package and a development version. The only changes allowed to be made on the release version are bug fixes and documentation improvements. This ensures that users will not encounter radical new behaviors in code obtained in the release version. All other changes such as enhancements or design changes are carried out on the development branch [34].

Approximately six weeks before a release, a major effort is taken to ensure that all packages on the development branch are coordinated and work well together. During that period extensive testing is carried out through peer review amongst the Bioconductor core. At release time all packages on the development branch that are included in the release change modes and are now released packages. Previous versions of these packages are deprecated in favor of the newly released versions. Simultaneously, a new development branch is made and the developers start to work on packages in the new branch. Note that these version-related administrative operations occur with little impact on developers. The release manager is responsible for package snapshot and file version modifications. The developers' source code base is fairly simple, and need not involve retention of multiple copies of any source code files, even though two versions are active at all times.

We would also like to point out that there are compelling arguments that can be made in favor of choosing different paradigms for software development and deployment. We are not attempting at this juncture to convince others to distribute software in this way, but rather elucidating our views and the reasons that we made our choice. Under a different set of conditions, or with different goals, it is entirely likely that we would have chosen a different model.

#### Special concerns

We now consider four specific challenges that are raised by research in computational biology and bioinformatics: reproducibility, data evolution and complexity, training users, and responding to user needs.

##### Reproducible research

We would like to address the reproducibility of published work in CBB. Reproducibility is important in its own right, and is the standard for scientific discovery. Reproducibility is an important step in the process of incremental improvement or refinement. In most areas of science researchers continually improve and extend the results of others but for scientific computation this is generally the exception rather than the rule.

Buckheit and Donoho [35], referring to the work and philosophy of Claerbout, state the following principle: "An article about computational science in a scientific publication is not the scholarship itself, it is merely advertising of the scholarship. The actual scholarship is the complete software development environment and that complete set of instructions that generated the figures."

There are substantial benefits that will come from enabling authors to publish not just an advertisement of their work but rather the work itself. A paradigm that fundamentally shifts publication of computational science from an advertisement of scholarship to the scholarship itself will be a welcome addition. Some of the concepts and tools that can be used in this regard are contained in [36,37].

When attempting to re-implement computational methodology from a published description many difficulties are encountered. Schwab *et al. *[38] make the following points:

"Indeed the problem occurs wherever traditional methods of scientific publication are used to describe computational research. In a traditional article the author merely outlines the relevant computations: the limitations of a paper medium prohibit complete documentation including experimental data, parameter values and the author's programs. Consequently, the reader has painfully to re-implement the author's work before verifying and utilizing it.... The reader must spend valuable time merely rediscovering minutiae, which the author was unable to communicate conveniently."

The development of a system capable of supporting the convenient creation and distribution of reproducible research in CBB is a massive undertaking. Nevertheless, the Bioconductor project has adopted practices and standards that assist in partial achievement of reproducible CBB.

Publication of the data from which articles are derived is becoming the norm in CBB. This practice provides one of the components needed for reproducible research - access to the data. The other major component that is needed is access to the software and the explicit set of instructions or commands that were used to transform the data to provide the outputs on which the conclusions of the paper rest. In this regard publishing in CBB has been less successful. It is easy to identify major publications in the most prestigious journals that provide sketchy or indecipherable characterizations of computational and inferential processes underlying basic conclusions. This problem could be eliminated if the data housed in public archives were accompanied by portable code and scripts that regenerate the article's figures and tables.

The combination of R's well-established platform independence with Bioconductor's packaging and documentation standards leads to a system in which distribution of data with working code and scripts can achieve most of the requirements of reproducible and replayable research in CBB. The steps leading to the creation of a table or figure can be clearly exposed in an Sweave document. An R user can export the code for modification or replay with variations on parameter settings, to check robustness of the reported calculations or to explore alternative analysis concepts.

Thus we believe that R and Bioconductor can provide a start along the path towards generally reproducible research in CBB. The infrastructure in R that is used to support replayability and remote robustness analysis could be implemented in other languages such as Perl [39] and Python [40]. All that is needed is some platform-independent format for binding together the data, software and scripts defining the analysis, and a document that can be rendered automatically to a conveniently readable account of the analysis steps and their outcomes. If the format is an R package, this package then constitutes a single distributable software element that embodies the computational science being published. This is precisely the compendium concept espoused in [36].

##### Dynamics of biological annotation

Metadata are data about data and their definition depends on the perspective of the investigator. Metadata for one investigator may well be experimental data for another. There are two major challenges that we will consider. First is the evolutionary nature of the metadata. As new experiments are done and as our understanding of the biological processes involved increases the metadata changes and evolves. The second major problem that concerns metadata data is its complexity. We are trying to develop software tools that make it easier for data analysts and researchers to use the existing metadata appropriately.

The constant changing and updating of the metadata suggests that we must have a system or a collection process that ensures that any metadata can be updated and the updates can be distributed. Users of our system will want access to the most recent versions. Our solution has been to place metadata into R packages. These packages are built using a semi-automatic process [41] and are distributed (and updated) using the package distribution tools developed in the *reposTools *package. There is a natural way to apply version numbers so users can determine if their data are up to date or if necessary they can obtain older versions to verify particular analyses. Further, users can synchronize a variety of metadata packages according to a common version of the data sources that they were constructed from.

There are a number of advantages that come from automating the process of building data packages. First, the modules are uniform to an extent that would not be possible if the packages were human written. This means that users of this technology need only become acquainted with one package to be acquainted with all such packages. Second, we can create many packages very quickly. Hence the labor savings are substantial. For microarray analyses all data packages should have the same information (chromosomal location, gene ontology categories, and so on). The only difference between the packages is that each references only the specific set of genes (probes) that were assayed. This means that data analysts can easily switch from one type of chip to another. It also means that we can develop a single set of tools for manipulating the metadata and improvements in those tools are available to all users immediately. Users are free to extend data packages with data from other, potentially proprietary, sources.

Treating the data in the same manner that we treat software has also had many advantages. On the server side we can use the same software distribution tools, indicating updates and improvements with version numbering. On the client side, the user does not need to learn about the storage or internal details of the data packages. They simply install them like other packages and then use them.

One issue that often arises is whether one should simply rely on online sources for metadata. That is, given an identifier, the user can potentially obtain more up-to-date information by querying the appropriate databases. The data packages we are proposing cannot be as current. There are, however, some disadvantages to the approach of accessing all resources online. First, users are not always online, they are not always aware of all applicable information sources and the investment in person-time to obtain such information can be high. There are also issues of reproducibility that are intractable as the owners of the web resources are free to update and modify their offerings at will. Some, but not all, of these difficulties can be alleviated if the data are available in a web services format.

Another argument that can be made in favor of our approach, in this context, is that it allows the person constructing the data packages to amalgamate disparate information from a number of sources. In building metadata packages for Bioconductor, we find that some data are available from different sources, and under those circumstances we look for consensus, if possible. The process is quite sophisticated and is detailed in the *AnnBuilder *package and paper [41].

##### Training

Most of the projects in CBB require a combination of skills from biology, computer science, and statistics. Because the field is new and there has been little specialized training in this area it seems that there is some substantial benefit to be had from paying attention to training. From the perspective of the Bioconductor project, many of our potential users are unfamiliar with the R language and generally are scientifically more aligned with one discipline than all three. It is therefore important that we produce documentation for the software modules that is accessible to all. We have taken a two-pronged approach to this, we have developed substantial amounts of course material aimed at all the constituent disciplines and we have developed a system for interactive use of software and documentation in the form of vignettes and more generally in the form of navigable documents with dynamic content.

Course materials have been developed and refined over the past two to three years. Several members of the Bioconductor development team have taught courses and subsequently refined the material, based on success and feedback. The materials developed are modular and are freely distributed, although restrictions on publication are made. The focus of the materials is the introduction and use of software developed as part of the Bioconductor project, but that is not a requirement and merely reflects our own specific purposes and goals.

In this area we feel that we would benefit greatly from contributions from those with more experience in technical document authoring. There are likely to be strategies, concepts and methodologies that are standard practice in that domain that we are largely unaware of. However, in the short term, we rely on the students, our colleagues and the users of the Bioconductor system to guide us and we hope that many will contribute. Others can easily make substantial contributions, even those with little or no programming skills. What is required is domain knowledge in one field of interest and the recognition of a problem that requires additional domain knowledge from another of the fields of interest.

Our experience has been that many of these new users often transform themselves into developers. Thus, our development of training materials and documentation needs to pay some attention to the needs of this group as well. There are many more software components than we can collectively produce. Attracting others to collaboratively write software is essential to success.

##### Responding to user needs

The success of any software project rests on its ability to both provide solutions to the problems it is addressing and to attract a user community. Perhaps the most effective way of addressing user needs is through an e-mail help list and one was set up as soon as the project became active. In addition it is important to keep a searchable archive available so that the system itself has a memory and new users can be referred there for answers to common questions. It is also important that members of the project deal with bug reports and feature requests through this public forum as it both broadcasts their intentions and provides a public record of the discussion. Our mailing list (mailto:bioconductor@stat.math.ethz.ch) has been successful: there are approximately 800 subscribers and about 3,000 email messages per year.

Attracting a user community itself requires a method of distributing the software and providing sufficient training materials to allow potential users to explore the system and determine whether it is sufficient for their purposes. An alternate approach would be to develop a graphical user interface (GUI) that made interactions with the system sufficiently self-explanatory that documentation was not needed. We note that this solution is generally more applicable to cases where the underlying software tasks are well defined and well known. In the present case, the software requirements (as well as the statistical and biological requirements) are constantly evolving. R is primarily command-line oriented and we have chosen to follow that paradigm at least for the first few years of development. We would of course welcome and collaborate with those whose goal was in GUI development but our own forays into this area are limited to the production of a handful of widgets that promote user interaction at specific points.

Users have experienced difficulties downloading and installing both R and the Bioconductor modules. Some of these difficulties have been caused by the users' local environments (firewalls and a lack of direct access to the internet), and some by problems with our software (bugs) which arise in part because it is in general very difficult to adequately test software that interacts over the internet. We have, however, managed to help every user, who was willing to persist, get both R and Bioconductor properly installed. Another substantial difficulty that we had to overcome was to develop a system that allowed users to download not just the software package that they knew they wanted, but additionally, and at the same time, all other software packages that it relies on. With Bioconductor software there is a much larger inter-reliance on software packages (including those that provide machine learning, biological metadata and experimental data) than for most other uses of R and the R package system. The package, reposTools contains much of the necessary infrastructure for handling these tasks. It is a set of functions for dealing with R package repositories which are basically internet locations for collections of R packages.

Once the basic software is installed, users will need access to documentation such as the training materials described above and other materials such as the vignettes, described in a previous section. Such materials are most valuable if the user can easily obtain and run the examples on their own computer. We note the obvious similarity with this problem and that described in the section on reproducible research. Again, we are in the enjoyable situation of having a paradigm and tools that can serve two purposes.

### Other open-source bioinformatics software projects

The Open Bioinformatics Foundation supports projects similar to Bioconductor that are nominally rooted in specific programming languages. BioPerl [42], BioPython [43] and BioJava [44] are prominent examples of open-source language-based bioinformatics projects. The intentions and design methodologies of the BioPerl project have been lucidly described by Stajich and colleagues [45].

#### BioPerl

In this section we consider commonalities and differences between BioPerl and Bioconductor. Both projects have commitments to open source distribution and to community-based development, with an identified core of developers performing primary design and maintenance tasks for the project. Both projects use object-oriented programming methodology, with the intention of abstracting key structural and functional features of computational workflows in bioinformatics and defining stable application programming interfaces (API) that hide implementation details from those who do not need to know them. The toolkits are based on highly portable programming languages. These languages have extensive software resources developed for non-bioinformatic purposes. The repositories for R (Comprehensive R Archive Network, CRAN) and Perl (Comprehensive Perl Archive Network, CPAN) provide mirrored WWW access to structured collections of software modules and documents for a wide variety of workflow elements. Development methodologies targeted at software reuse can realize large gains in productivity by establishing interfaces to existing CPAN or CRAN procedures instead of reimplementing such procedures. For reuse to succeed, the maintainer of the external resource must commit to stability of the resource API. Such stability tends to be the norm for widely-used modules. Finally, both languages have considerable interoperability infrastructure. One implication is that each project can use software written in unrelated languages. R has well-established interfaces to Perl, Python, Java and C. R's API allows software in R to be called from other languages, and the *RSPerl *package [46] facilitates direct calls to R from Perl. Thus there are many opportunities for symbiotic use of code by Bioconductor and BioPerl developers and users. The following script illustrates the use of BioPerl in R.

> library(RSPerl)

> .PerlPackage("Bio::Perl")

> x <- .Perl("get_sequence", "swiss",

    "ROA1_HUMAN")

> x$division()

[1] "HUMAN"

> x$accession()

[1] "P09651"

> unlist(x$get_keywords())

[1] "Nuclear protein" "RNA-binding"

[3] "Repeat" "Ribonucleoprotein"

[5] "Methylation" "Transport"

...

The .PerlPackage command brings the BioPerl modules into scope. .Perl invokes the BioPerl get_sequence subroutine with arguments "swiss" and "ROA1_HUMAN". The resulting R object is a reference to a perl hash. RSPerl infrastructure permits interrogation of the hash via the $ operator. Note that *RSPerl *is not a Bioconductor-supported utility, and that installation of the BioPerl and *RSPerl *resources to allow interoperation can be complicated.

Key differences between the Bioconductor and BioPerl projects concern scope, approaches to distribution, documentation and testing, and important details of object-oriented design.

##### Scope

BioPerl is clearly slanted towards processing of sequence data and interfacing to sequence databases, with support for sequence visualization and queries for external annotation. Bioconductor is slanted towards statistical analysis of microarray experiments, with major concerns for array preprocessing, quality control, within- and between-array normalization, binding of covariate and design data to expression data, and downstream inference on biological and clinical questions. Bioconductor has packages devoted to diverse microarray manufacturing and analysis paradigms and to other high-throughput assays of interest in computational biology, including serial analysis of gene expression (SAGE), array comparative genomic hybridization (arrayCGH), and proteomic time-of-flight (SELDI-TOF) data. We say the projects are 'slanted' towards these concerns because it is clear that both projects ultimately aim to support general research activities in computational biology.

##### Distribution, documentation and testing

BioPerl inherits the distribution paradigm supported by CPAN. Software modules can be acquired and installed interactively using, for example perl -MCPAN -e shell. This process supports automated retrieval of requested packages and dependencies, but is not triggered by runtime events. Bioconductor has extended the CRAN distribution functionalities so that packages can be obtained and installed 'just in time', as required by a computational request. For both Perl and R, software modules and packages are structured collections of files, some of which are source code, some of which are documents about the code. The relationship between documentation and testing is somewhat tighter in Bioconductor than in BioPerl. Manual pages and vignettes in Bioconductor include executable code. Failure of the code in a man page or vignette is a quality-control event; experimentation with executable code in manual pages (through the example function of R) is useful for learning about software behavior. In Perl, tests occupy separate programs and are not typically integrated with documentation.

##### Details of object-oriented procedure

Both R and Perl are extensible computer languages. Thus it is possible to introduce software infrastructure supporting different approaches to object-oriented programming (OOP) in various ways in both languages.

R's core developers have provided two distinct approaches to OOP in R. These approaches are named S3 and S4. In S3, any object can be assigned to a class (or sequence of classes) simply by setting the class name as the value of the object's class attribute. Class hierarchies are defined implicitly at the object level. Generic methods are defined as ordinary functions and class-specific methods are dispatched according to the class of the object being passed as an argument. In S4, formal definition of class structure is supported, and class hierarchy is explicitly defined in class definitions [12]. Class instances are explicitly constructed and subject to validation at time of construction. Generic methods are non-standard R functions and metadata on generic methods is established at the package level. Specific methods are dispatched according to the class signature of the argument list (multiple dispatch). Overall, the OOP approach embodied in S4 is closer to Dylan or Scheme than to C++ or Java. Bioconductor does not require specific OOP methodology but encourages the use of S4, and core members have contributed special tools for the documentation and testing of S4 OOP methods in R.

OOP methodology in Perl has a substantial history and is extensively employed in BioPerl. The basic approach to OOP in Perl seems to resemble S3 more than S4, in that Perl's bless operation can associate any perl data instance with any class. The CPAN Class::Multimethod module can be used to allow multiple dispatch behavior of generic subroutines. The specific classes of objects identified in BioPerl are targeted at sequence data (Seq, LocatableSeq, RelSegment are examples), location data (Simple, Split, Fuzzy), and an important class of objects called interface objects, which are classes whose names end in 'I'. These objects define what methods can be called on objects of specified classes, but do not implement any methods.

#### BioJava, BioPython, GMOD and MOBY

Other open bioinformatics projects have intentions and methods that are closely linked with those of Bioconductor.

BioJava [44] provides Dazzle, a servlet framework supporting the Distributed Annotation System specification for sharing sequence data and metadata. Version 1.4 of the BioJava release includes java classes for general alphabets and symbol-list processing, tools for parsing outputs of blast-related analyses, and software for constructing and fitting hidden Markov models. In principle, any of these resources could be used for analysis in Bioconductor/R through the *SJava *interface [46].

BioPython [43] provides software for constructing python objects by parsing output of various alignment or clustering algorithms, and for a variety of downstream tasks including classification. BioPython also provides infrastructure for decomposition of parallelizable tasks into separable processes for computation on a cluster of workstations.

The Generic Model Organism Database (GMOD) project targets construction of reusable components that can be used to reproduce successful creation of open and widely accessible databases of model organisms (for example, worm, fruitfly and yeast). The main tasks addressed are genome visualization and annotation, literature curation, biological ontology activities, gene expression analysis and pathway visualization and annotation.

BioMOBY [47] provides a framework for developing and cataloging web services relevant to molecular biology and genomics. A basic aim is to provide a central registry of data, annotation or analysis services that can be used programmatically to publish and make use of data and annotation resources pertinent to a wide variety of biological contexts.

As these diverse projects mature, particularly with regard to interoperability, we expect to add infrastructure to Bioconductor to simplify the use of these resources in the context of statistical data analysis. It is our hope that the R and Bioconductor commitments to interoperability make it feasible for developers in other languages to reuse statistical and visualization software already present and tested in R.

### Using Bioconductor (example)

Results of the Bioconductor project include an extensive repository of software tools, documentation, short course materials, and biological annotation data at [1]. We describe the use of the software and annotation data by description of a concrete analysis of a microarray archive derived from a leukemia study.

Acute lymphocytic leukemia (ALL) is a common and difficult-to-treat malignancy with substantial variability in therapeutic outcomes. Some ALL patients have clearly characterized chromosomal aberrations and the functional consequences of these aberrations are not fully understood. Bioconductor tools were used to develop a new characterization of the contrast in gene expression between ALL patients with two specific forms of chromosomal translocation. The most important tasks accomplished with Bioconductor employed simple-to-use tools for state-of-the-art normalization of hundreds of microarrays, clear schematization of normalized expression data bound to detailed covariate data, flexible approaches to gene and sample filtering to support drilling down to manageable and interpretable subsets, flexible visualization technologies for exploration and communication of genomic findings, and programmatic connection between expression platform metadata and biological annotation data supporting convenient functional interpretation. We will illustrate these through a transcript of the actual command/output sequence. More detailed versions of some of the processing and analysis activities sketched here can be found in the vignettes from the *GOstats *package.

The dataset is from the Ritz laboratory at the Dana Farber Cancer Institute [48]. It contains data from 128 patients with ALL. Two subgroups are to be compared. The first group consists of patients with a translocation between chromosomes 4 and 11 (labeled ALL1/AF4). The second group consists of patients with a translocation between chromosomes 9 and 22 (labeled BCR/ABL). These conditions are mutually exclusive in this dataset.

The Affymetrix HGu95Av2 platform was used, and expression measures were normalized using *gcrma *from the *affy *package. The output of this is an object of class *exprSet *which can be used as input for other functions. The package *hgu95av2 *provides biological metadata including mappings from the Affymetrix identifiers to GO, chromosomal location, and so on. These data can, of course be obtained from many other sources, but there are some advantages to having them as an R package.

After loading the appropriate packages we first subset the ALL exprSet to extract those samples with the covariates of interest. The design of the exprSet class includes methods for subsetting both cases and probes. By using the square-bracket notation on ALL, we derive a new exprSet with data on only the desired patients.

> data("ALL")

> eset <- ALL[, ALL$mol %in%

c("BCR/ABL", "ALL1/AF4")]

**Figure 1:**
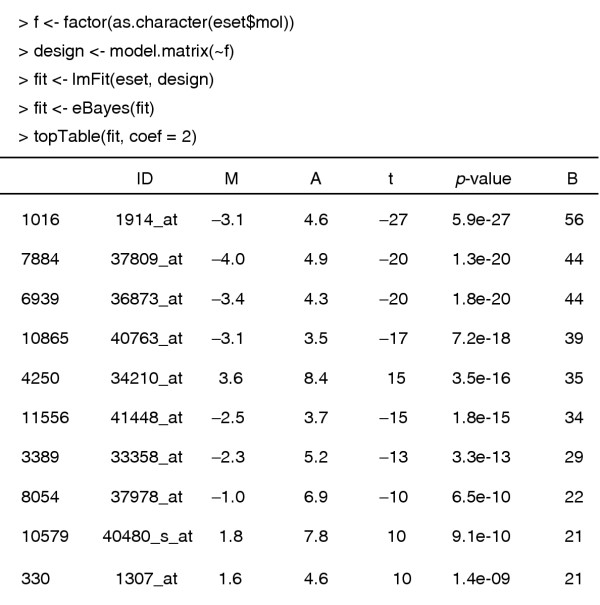
Limma analysis of the ALL data. The leftmost numbers are row indices, ID is the Affymetrix HGU95av2 accession number, M is the log ratio of expression, A is the log average expression, and B is the log odds of differential expression.

Next we find genes which are differentially expressed between the ALL1/AF4 and BCR/ABL groups. We use the function lmFit from the *limma *package, which can assess differential expression between many different groups and conditions simultaneously. The function lmFit accepts a model matrix which describes the experimental design and produces an output object of class MArrayLM which stores the fitted model information for each gene. The fitted model object is further processed by the eBayes function to produce empirical Bayes test statistics for each gene, including moderated *t*-statistics, *p*-values and log-odds of differential expression. The log_2_-fold changes, average intensites and Holm-adjusted *p*-values are displayed for the top 10 genes (Figure 1).

We select those genes that have adjusted *p*-values below 0.05. The default method of adjusting for multiple comparisons uses Holm's method to control the family-wise error rate. We could use a less conservative method such as the false discovery rate, and the multtest package offers other possibilities, but for this example we will use the very stringent Holm method to select a small number of genes.

> selected <- p.adjust(fit$p.value[, 2])

< 0.05

> esetSel <- eset [selected, ]

**Figure 2:**
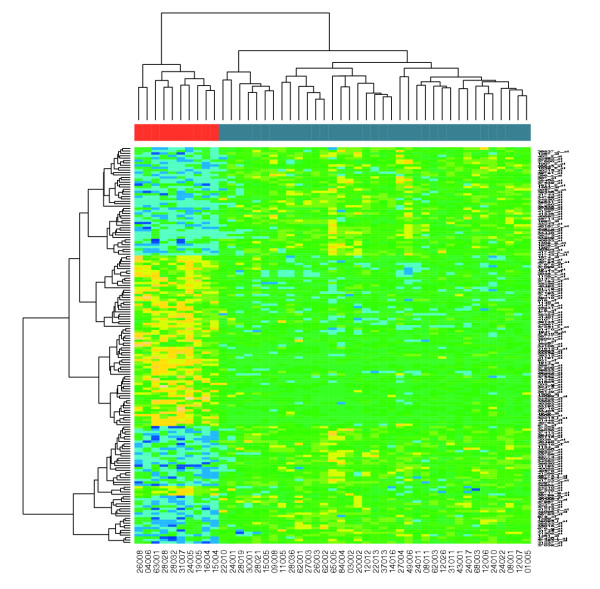
Heat map (produced by the Bioconductor function heatmap()) of the ALL leukemia data.

There are 165 genes selected for further analysis. A heat map produced by the heatmap function from R allows us to visualize the differential action of these genes between the two groups of patients. Note how the different software modules can be integrated to provide a very rich data-analysis environment. Figure 2 shows clearly that these two groups can be distinguished in terms of gene expression.

We can carry out many other tests, for example, whether genes encoded on a particular chromosome (or perhaps on a specific strand of a chromosome) are over-represented amongst those selected by moderated *t*-test. Many of these questions are normally addressed in terms of a hypergeometric distribution, but they can also be thought of as two-way or multi-way tables, and alternate statistical tests (all readily available in R) can be applied to the resulting data.

**Figure 3:**
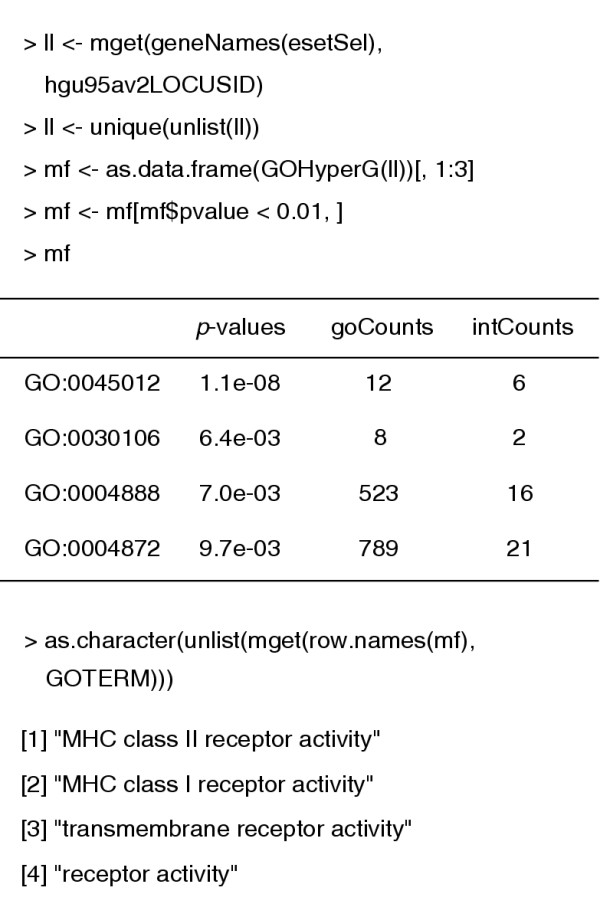
Hypergeometric analysis of molecular function enrichment of genes selected in the analysis described in Figure 1.

We turn our attention briefly to the use of the Gene Ontology (GO) annotation in conjunction with these data. We first identify the set of unique LocusLink identifiers among our selected Affymetrix probes. The function GOHyperG is found in the *GOstats *package. It carries out a hypergeometric test for an overabundance of genes in our selected list of genes for each term in the GO graph that is induced by these genes (Figure 3).

The smallest *p*-value found was 1.1e-8 and it corresponds to the term, "MHC class II receptor activity". We see that six of the 12 genes with this GO annotation have been selected. Had we used a slightly less conservative gene selection method then the number of selected genes in this GO annotation would have been even higher.

Reproducing the above results for any other species or chip for which an annotation package was available would require almost no changes to the code. The analyst need only substitute the references to the data package, *hgu95av2*, with those for their array and the basic principles and code are unchanged.

Similarly, substitution of other algorithms or statistical tests is possible as the data analyst has access to the full and complete source code. All tools are modifiable at the source level to suit local requirements.

## Conclusions

We have detailed the approach to software development taken by the Bioconductor project. Bioconductor has been operational for about three years now and in that time it has become a prominent software project for CBB. We argue that the success of the project is due to many factors. These include the choice of R as the main development language, the adoption of standard practices of software design and a belief that the creation of software infrastructure is an important and essential component of a successful project of this size.

The group dynamic has also been an important factor in the success of Bioconductor. A willingness to work together, to see that cooperation and coordination in software development yields substantial benefits for the developers and the users and encouraging others to join and contribute to the project are also major factors in our success.

To date the project provides the following resources: an online repository for obtaining software, data and metadata, papers, and training materials; a development team that coordinates the discussion of software strategies and development; a user community that provides software testing, suggested improvements and self-help; more than 80 software packages, hundreds of metadata packages and a number of experimental data packages.

At this point it is worth considering the future. While many of the packages we have developed have been aimed at particular problems, there have been others that were designed to support future developments. And that future seems very interesting. Many of the new problems we are encountering in CBB are not easily addressed by technology transfer, but rather require new statistical methods and software tools. We hope that we can encourage more statisticians to become involved in this area of research and to orient themselves and their research to the mixture of methodology and software development that is necessary in this field.

In conclusion we would like to note that the Bioconductor Project has many developers, not all of whom are authors of this paper, and all have their own objectives and goals. The views presented here are not intended to be comprehensive nor prescriptive but rather to present our collective experiences and the authors' shared goals. In a very simplified version these can be summarized in the view that coordinated cooperative software development is the appropriate mechanism for fostering good research in CBB.

## Figures and Tables

**Table 1:** Reasons for deciding to release software under an open-source license

To encourage reproducibility, extension and general adherence to the scientific method
To ensure that the code is open to public scrutiny and comment
To provide full access to algorithms and their implementation
To provide to users the ability to fix bugs without waiting for the developer, and to extend and improve the supplied software
To encourage good scientific computing and statistical practice by exhibiting fully appropriate tools and instruction
To provide a workbench of tools that allow researchers to explore and expand the methods used to analyze biological data
To ensure that the international scientific community is the owner of the software tools needed to carry out research
To lead and encourage commercial support and development of those tools that are successful
To promote reproducible research by providing open and accessible tools with which to carry out that research
